# Characterization of the complete chloroplast genome of *Paederia scandens* (Rubiaceae): a Chinese folk medicinal plant

**DOI:** 10.1080/23802359.2019.1691068

**Published:** 2019-11-18

**Authors:** Zheng-Hui Li, Ke Ye, Xiao Lv, Xian Zhang, Li-Zhen Ling, Hong-Lian Ai, Shu-Dong Zhang

**Affiliations:** a School of Pharmaceutical Sciences, South-Central University for Nationalities, Wuhan, China;; b School of Biological Sciences and Technology, Liupanshui Normal University, Liupanshui, China

**Keywords:** Chloroplast genome, medicinal plant, *Paederia scandens*, phylogenetic analysis

## Abstract

*Paederia scandens* has been used as a traditional medicine in Asian countries to treat jaundice, dysentery, and the pain of rheumatism. The complete chloroplast (cp) genome sequence of *P. scandens* was reported and characterized in this study. The cp genome is 153,626 bp in length, composed of a pair of 26,513 bp inverted repeat (IR) regions separated by a large single-copy (LSC) region of 83,712 bp and a small single-copy (SSC) region of 16,888 bp. There were 131 predicted genes (86 protein-coding genes, 37 tRNA genes, and 8 rRNA genes) in the genome, and the overall GC content of the genome is 37.7%. Phylogenetic analysis based on the cp genome data showed that *P. scandens* was sister to the clade formed by *Galium mollugo* and *G. aparine*.

*Paederia scandens* (Lour.) Merr. (Rubiaceae) has been traditionally used as medicinal herbs to treat the pain of rheumatism, jaundice, dysentery, dyspepsia, and abdominal mass in China, Japan, India, and Malaysia (Chen et al. 2007; Wang et al. 2014). As a Chinese folk medicine, *P. scandens* was reported to have antinociceptive, anticonvulsant, hepatoprotective, and anti-inflammatory activities (Zhu et al. 2012; Yang et al. 2013; Hou et al. 2014; Peng et al. 2015; Xiao et al. 2018). Some bioactive compounds including iridoids glycosides (IGS), flavonoids, and volatile oil have been identified in *P. scandens* (Kim et al. 2004; Wang et al. 2015). In this study, we aim to establish and characterize the complete chloroplast (cp) genome of *P*. *scandens*, and assess its phylogenetic position within Rubiaceae.

Fresh leaves of *P*. *scandens* were collected from Longping town of Jianshi county in Hubei province (N30°48′24″, E110°1′47″, 1,750 m). The voucher specimen (HSN12426) was deposited in the herbarium of South-Central University for Nationalities (HSN). The total DNA of *P*. *scandens* was extracted for sequencing on Illumina HiSeq 4000 Platform at the Beijing Novogene Bioinformatics Technology Co., Ltd. (Nanjing, China). About 2 Gb raw data were used to *de novo* assemble the complete cp genome using SPAdes (Bankevich et al. 2012). The complete genome sequence was annotated using Dual Organellar Genome Annotator (DOGMA) (Wyman et al. 2004) with manual adjustments. The sequence of cp genome was deposited in GenBank (accession number MN567112).

The complete cp genome of *P*. *scandens* was 153,626 bp in size. This genome was with a typical quadripartite structure, containing a small single-copy (SSC) region of 16,888 bp, a large single-copy (LSC) region of 83,712 bp and two inverted repeat (IR) regions of 26,513 bp. The GC content of this genome was 37.7%. There were a total of 131 genes including 86 protein-coding genes, 37 tRNA genes, and 8 rRNA genes in the genome. Most of the genes occurred in a single copy, while four rRNA genes (i.e. 4.5S, 5S, 16S, and 23S rRNA), seven tRNA genes (i.e. *trnA-UGC*, *trnI-CAU*, *trnI-GAU*, *trnL-CAA*, *trnN-GUU*, *trnR-ACG*, and *trnV-GAC*) and seven protein-coding genes (i.e. *rpl2*, *rpl23*, *ycf2*, *ycf15*, *ndhB*, *rps7*, and *rps12*) occurred in double. Among the 131 genes, 19 of them had one intron, and three had two introns (*clpP*, *rps12*, and *ycf3*).

To confirm the phylogenetic position of *P*. *scandens*, phylogenetic analysis was conducted based on the complete cp genomes of this species and other 16 species belonging to Rubiaceae, Gentianaceae, Gelsemiaceae, and Asclepiadaceae. The sequences were aligned with MAFFT (Katoh and Standley 2013). The maximum-likelihood (ML) and Bayesian inference (BI) phylogenetic trees were reconstructed using RAxML (Stamatakis 2014) and MrBayes (Ronquist et al. 2012). The ML and BI analyses generated the same tree topology (Figure 1). As shown in the phylogenetic tree (Figure 1), *P*. *scandens* was closely related to the genus *Galium* with 100% bootstrap and 1.0 posterior probability support, respectively. Our findings will provide a foundation for further investigation of cp genome evolution in *Paederia*, and also could be further applied for evolutionary and phylogenetic studies of *Paederia*, even Rubiaceae.

**Figure 1.**
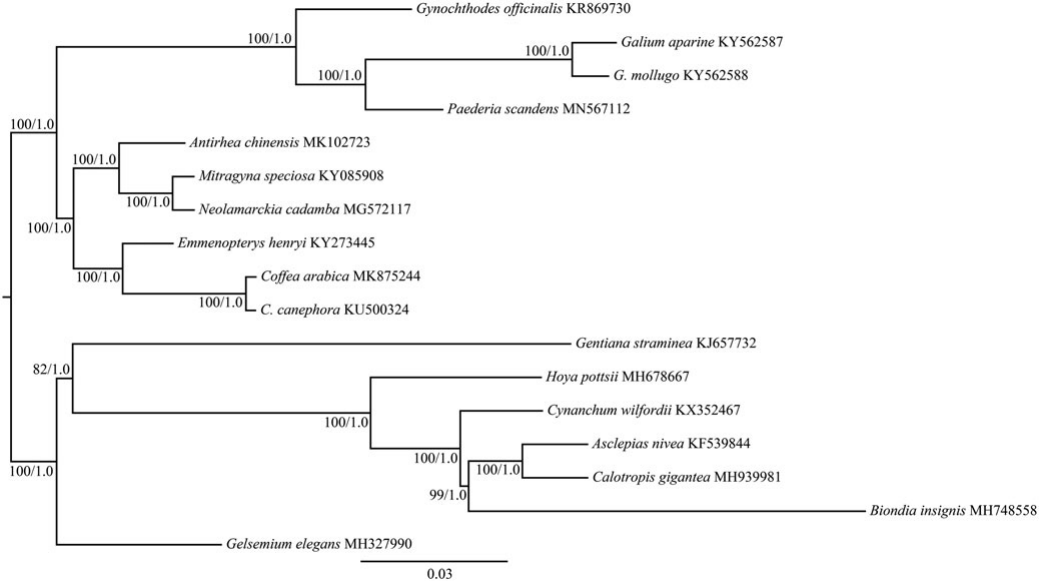
The maximum-likelihood (ML) tree of Rubiaceae inferred from the complete chloroplast genome sequences. Numbers at nodes correspond to ML bootstrap percentages (1,000 replicates) and Bayesian inference (BI) posterior probabilities.
